# Agreement, reliability and validity in 3 shoulder questionnaires in patients with rotator cuff disease

**DOI:** 10.1186/1471-2474-9-68

**Published:** 2008-05-15

**Authors:** Ole M Ekeberg, Erik Bautz-Holter, Einar K Tveitå, Anne Keller, Niels G Juel, Jens I Brox

**Affiliations:** 1 Department of Physical Medicine and Rehabilitation, Ullevaal University Hospital, Oslo, Norway; 2 Department of Orthopaedics, Section for Physical Medicine and Rehabilitation National Hospital, Oslo, Norway

## Abstract

**Background:**

Self-report questionnaires play an important role as outcome measures in shoulder research. Having an estimate of the measurement error of these questionnaires is of importance when assessing follow-up results after treatment and when planning intervention studies. The aim of this study was to cross-culturally adapt the Norwegian version of the OSS and WORC questionnaire and examine and compare agreement, reliability and construct validity of the disease-specific shoulder questionnaire WORC with two commonly used shoulder questionnaires, SPADI and OSS, in patients with rotator cuff disease.

**Methods:**

74 patients with rotator cuff disease were recruited from the outpatient clinic of the Physical Medicine and Rehabilitation Department at Ullevaal University Hospital in Oslo, Norway. A test-retest design was used, and the questionnaires were filled out by the patients at the clinic, with a one week interval between test administrations. Agreement (repeatability coefficient), reliability (ICC) and construct validity were examined and compared for WORC, SPADI and OSS.

**Results:**

Reliability analysis was restricted to the 55 patients (51 ± 10 yrs) who reported no change between test administrations according to scoring on a global scale. The agreement, reliability and construct validity was moderate for all three questionnaires with ICC ranging from 0.83 to 0.85, repeatability coefficient from 16.1 to 19.7 and Spearman rank correlations between total scores from r = 0.57 to 0.69. There was a lower degree of floor and ceiling effects in SPADI compared to WORC and OSS.

**Conclusion:**

We conclude that the agreement and reliability of the three shoulder questionnaires examined, WORC index, SPADI and OSS are acceptable and that differences between scores were small. The Norwegian version of the questionnaires is acceptable for assessing Norwegian-speaking patients with rotator cuff disease. The moderate agreement and construct validity should be taken into consideration when assessing follow-up results after treatment and in the planning of prospective studies.

## Background

Assessment of patients with shoulder pain in a standardized and validated way has been recognized as being important in research [1]. In the past decades, a number of self-report questionnaires have been developed to assess shoulder pain and disability from the patient's perspective. Choosing a shoulder questionnaire for use in a clinical intervention study is, however, not an easy task according to current evidence in the literature. A recent review suggested that the choice should be based on different factors such as the study group, clinimetric properties of the questionnaires and practical considerations in terms of ease of scoring and time to complete a questionnaire [2]. In this review the authors found that some questionnaires have been tested more extensively than others, although many of the validation studies are hampered with methodological limitations. It has been argued that disease-specific questionnaires possess better clinimetric properties than generic questionnaires [3,4]. Recently two different rotator cuff specific questionnaires have been developed, the Western Ontario Rotator Cuff index (WORC) and the Rotator Cuff Quality of life questionnaire (RC-QOL) [3,5]. A few studies have compared the clinimetric properties of WORC to general shoulder questionnaires, but none have found convincing evidence for claiming this new questionnaire to be better suited for patients with rotator cuff disease than the general shoulder questionnaires [6-9].

Estimating measurement error in these instruments is important because of the direct impact on reliability, effect size, responsiveness and sample size calculations [10,11]. Generally, a reduction of measurement error will result in a higher reliability, thereby reducing the sample size required to detect significant treatment effects. Quantifying and interpreting measurement error and reliability has proven difficult in the biomedical literature [12,13]. There is an important distinction between reliability and agreement [14]. Reliability concerns the consistency of the positions or rank of individuals in the group relative to others and reflects the instruments ability to discriminate between subjects in a population sample. The Intraclass Correlation Coefficient (ICC) is the most appropriate reliability parameter for measurements on a continuous scale. Agreement parameters assess how close the results of the repeated measures are. Agreement parameters are preferred if the aim is to measure change in health status [14]. It is therefore important that both reliability and agreement is examined when assessing measurement properties in an instrument [13].

In clinical studies, shoulder questionnaires are often used to evaluate change between groups after an intervention. Thus, in these circumstances the agreement parameters are of most importance. Yet, there have been no reports of the measurement error of WORC in the literature. The developers of WORC describe a high reliability in WORC, with an ICC of 0.96 [3]. Most general shoulder questionnaires have been assessed in populations with a variety of causes of shoulder pain, and little is known about the measurement properties in specific shoulder conditions. Investigating the properties of an instrument in the sample and under the condition of intended use is important [15].

The choice of which questionnaire to use is becoming a major difficulty. Quality criteria have been proposed [16]. By example, a high Cronbach's alpha, which is considered an appropriate measure of internal consistency, indicates strong correlation between the items in a scale, which makes summarizing of the items supported. Floor and ceiling effects are considered to be present if more than 15% of the respondents achieved the lowest or highest possible score, respectively [17]. Construct validity refers to the extent which scores on a particular instrument relate to another instrument [18]. We hypothesized that the three shoulder questionnaires should be highly correlated in order to be used interchangeably.

The aim of the present study was cross-culturally adapt WORC and Oxford Shoulder score (OSS) for use in Norwegian-speaking patients and to evaluate agreement, reliability, floor and ceiling effects, internal consistency, and construct validity for these questionnaires and the Shoulder Pain and Disability Index (SPADI), in a population of patients with rotator cuff disease.

## Methods

This prospective methodological study was undertaken as a two-step process. First, the OSS and the WORC index were translated and adapted into Norwegian; second the three questionnaires were evaluated in a population of patients with rotator cuff disease.

### Questionnaires

The Western Ontario Rotator Cuff (WORC) index is a self-report questionnaire developed to measure health related quality of life in patients with rotator cuff disease [3]. WORC consists of 21 items in 5 domains: physical symptoms (6 items), sports and recreation (4 items), work (4 items), lifestyle (4 items) and emotions (3 items). Each question is scored on a 100 mm VAS scale and summed to a total score of maximally 2100, with a higher score indicating a reduced quality of life. A percentage score ranging from 0 (worst possible) to 100 (best possible) is used as advocated by its developers.

Oxford Shoulder Score (OSS) is a self-report questionnaire developed for patients having shoulder disease other than instability, and consists of 12 questions about pain and disability [19]. Respondents report their pain or difficulty in completing a task by circling a number from 1 to 5 with verbal anchors following each number. All items are summed up to a total score ranging from 12 to 60. To allow scores to be easily compared, the OSS total sum score was converted to range from 0 to 100. In the original publication of OSS, all respondents were asked to consider their shoulder for the last 4 weeks when completing the questionnaire. In order to compare the questionnaires, this was revised in the present study to yield the most recent week, to parallel the other questionnaires in the study.

Shoulder Pain and Disability Index (SPADI) is a self-report questionnaire for patients with shoulder pain and consists of 13 questions divided in two domains: pain (5 items) and disability (8 items) [20]. The questions are scored on VAS scales from 0 (best) to 11(worst) and summed up to a domain score. Each domain score is equally weighted, then added, for a total percentage score ranging from 0 to 100. A higher score indicates a worse shoulder pain and function.

### Translation

A culturally adapted Norwegian version of SPADI already exists. Translation of OSS and WORC was done according to the guidelines in the literature [21,22]. Forward translation of WORC from English to Norwegian was done by four bilingual medical doctors, one physiotherapist and one professional translator, all with Norwegian as their native language. Forward translation of OSS was done by one bilingual physiotherapist, a medical doctor and a professional translator. The translations were done independently of each other and then compared. Inconsistencies were resolved in a consensus meeting. The first Norwegian versions of both questionnaires were then back-translated into English by a professional translator and a medical doctor, both native English speakers. The back-translated versions were then reviewed and the final Norwegian version of the questionnaires was agreed upon in a consensus meeting.

### Patient selection

74 patients with rotator cuff disease were prospectively recruited from the outpatient clinic of the Physical Medicine and Rehabilitation Department at Ullevaal University Hospital in Oslo, Norway. Inclusion criteria consisted of a normal passive glenohumeral joint range of motion, pain on abducting the affected shoulder, pain on at least 2 of 3 isometric tests of abduction, external rotation and internal rotation and positive Hawkins test [23]. Patients with full-thickness rotator cuff rupture were included if they fulfilled the above mentioned inclusion criteria. Patients were excluded if they were previously operated in the painful shoulder, had clinical and radiological findings indicating glenohumeral joint pathology or had clinical signs of a cervical syndrome. All patients gave their informed consent and received oral and written information about the project. The Regional Committee for Medical Research Ethics in Norway approved the study.

### Procedures

The WORC index, OSS and SPADI were filled out by the patients at the outpatient clinic two times with a one week interval between administrations. The length of the test-retest interval was chosen because the patient's condition was unlikely to change substantially during this time interval, yet the time period was long enough for patients to essentially forget their initial response. Patients also answered a comprehensive questionnaire covering socio-demographic data and a global question of change in shoulder condition between visits ranging from -9 (maximum deterioration) to 9 (maximum improvement) [24]. All patients received a brief introduction to the questionnaires, but did not receive help filling in the questionnaires.

### Statistical analysis

The sample size was based on the general recommendations of Altman of at least 50 subjects in a methods comparison study [25].

For socio-demographic data and the domain and total sum scores of the questionnaires, the mean and the standard deviation (SD) or frequencies were calculated for numerical and categorical variables, respectively. Minimum and maximum scores for individual items, domain and total scores where examined for possible floor or ceiling effects. Floor or ceiling effects were considered to be present if more than 15% of respondents achieved the lowest or highest possible score, respectively [17].

### Agreement and reliability

Different statistical measures were used to examine agreement and reliability of the questionnaires. First, the change in mean scores between the test and retest was calculated. Differences between test and retest scores were compared by paired t-tests for each scale to assess any systematic differences between test and retest administrations. The percent identical scores on test and retest were examined for individual items, domain and total scores. Intraclass correlation coefficient (ICC) was used to assess reliability. The ICC can range between 0.00 (representing a totally unreliable measurement) and 1.00 (implying perfect reliability). Several forms of ICC exist [26]. In the present study a two-way random effects model single measure reliability (2,1) was used. ICC (2,1) was calculated with confidence intervals for individual items, domain score and total score for each questionnaire. The use of weighted Kappa is recommended for analysis of reliability in ordinal scales [16], but was omitted from the analysis because the weighted Kappa will be identical to the ICC when the most frequently used weighting scheme, the quadratic weight, is used [27].

Several agreement parameters can be found in the literature. Most references describes the use of the standard error of measurement (SEM) which is defined by *SEM *= σ *(1-ICC) *where σ is the pooled standard deviation of test and retest scores [12,28]. Since different forms of the ICC may affect the size of SEM, an alternative way of calculating the SEM by using the square root of the mean square error term of the analysis of variance (ANOVA) has been recommended [13]. SEM can be calculated as SEM_agreement _or SEM_consistency_[14]. SEM_agreement _take the systematic difference between test and retest into account while the SEM_consistency _ignore systematic differences. Bland and Altman recommend estimating the repeatability coefficient [29]. The repeatability coefficient is calculated by multiplying the within-subject standard deviation (S_w_) with 1.96√2. Two readings by the same method will be within the repeatability coefficient for 95% of subjects. S_w _is calculated by extracting the square root of the residual mean square, using one-way analysis of variance with subjects as the factor and equals SEM_agreement_. The repeatability coefficient defines the smallest detectable difference (SDD) between two measurements on the same individual. In the present study agreement was estimated using the repeatability coefficient. In addition, plots of the difference between the first and the second response on the questionnaires against a mean of the sum scores were constructed according to Bland and Altman's recommendations to get a graphical expression of the agreement. The standard deviation of the difference was multiplied by 2 and subtracted or added to the mean difference to create the 95% limits of agreement (LOA), which were drawn as lines in the plots. Internal consistency of the questionnaires was calculated by Cronbach's alpha for domain score, total score and by exploring the effect of deleting single items in the analysis.

### Validity

In order to evaluate the construct validity of the Norwegian version of the OSS and WORC, the Spearman rank correlation were calculated between total and domain scores of the OSS, WORC and SPADI. A priori we believed that there would be a high positive correlation among the questionnaires total sum scores as they were likely to measure the same constructs. We expected that the emotion domain of WORC to be only moderately correlated with SPADI and OSS scores. This hypothesis was based on a theory that WORC emotions domain measures another construct than pain and function.

Limits of Agreement (LOA) plots were created by using MedCalc for Windows, version 9.1.0.1 (MedCalc Software, Mariakerke, Belgium). All other statistics were analyzed by SPSS 13.0 (Statistical Software Package of the Social Sciences, Chicago, Illinois).

## Results

### Translation

The forward and back translation of OSS revealed no difficulties. Item 17 in WORC (How much difficulty do you have "roughhousing or horsing around" with family or friends?) and item 20 (How "down in the dumps" or depressed do you feel because of your shoulder?) needed rephrasing to reach an acceptable Norwegian translation. Other minor discrepancies were solved in the consensus meetings. The Norwegian versions of OSS and WORC index were pre-tested on 10 patients with shoulder pain. All items were well comprehended and no further changes were needed. Patients found the OSS easy to respond to. There were a few patients complaining of difficulties in answering item 8 (How much difficulties do you experience doing push-ups or other strenuous exercises because of your shoulder?) and item 9 (How much has your shoulder affected your ability to throw hard or far?) in WORC because they never did strenuous shoulder activities like push-ups or had never tried to throw hard or far. All patients were encouraged to read the "instructions to patients" in WORC, which states "if an item does not pertain to you or you have not experienced it in the past week, please make your "best guess" as to which response would be the most accurate". Permission to use the final Norwegian version of OSS and WORC was granted from the developers of the questionnaires.

### Agreement and reliability

Fifty-five of the 74 patients reported on the global score that their shoulder condition had not changed between administrations of the questionnaires, and scores for these 55 patients were used in the reliability analysis. The mean age of the 55 patients (19 male, 34%; 36 female, 66%) was 51 years (range 31–80, SD 10). All patients had experienced shoulder pain for more than 2 months (range 2–24 months). Median time between administrations was 7 days (range 5–11 days). There were no statistically significant differences in total scores between the first and second questionnaire administrations (Table 1). Table 2 shows the descriptive data, the percent identical responses, and ICC for each domain and total score. For OSS there was a high floor effect (score = 1) for 6 items, highest for item 4 (Have you been able to use knife and fork – at the same time?) with 76%. There was a moderate ceiling effect (score = 5) with 2 items with more than 15% of responses in the maximum score and highest for item 12 (Have you been troubled by pain from your shoulder in bed at night?) with 46%. The SPADI showed a moderate floor effect (score = 0) for 3 items, highest for item 10 (difficulty in putting on your pants?) with 23.6%. There was no evidence of a ceiling effect in SPADI (score = 11). WORC index was recoded from 0–100 to 1–10 in order to examine floor, ceiling and percent identical responses. WORC items showed a moderate floor effect (score = 1) for four items and highest for item 6 (How much discomfort do you experience in the muscles of your neck because of your shoulder?) with 23.6%. There was a moderate ceiling effect (score = 10) in 6 items in WORC, highest for item 9 (How much has your shoulder affected your ability to throw hard or far?) with 43.6%. There were no floor or ceiling effects in domain or total sum scores of any of the questionnaires. As expected the percent identical items on test and retest were highest in OSS with fewest answer categories and a possibility of a higher degree of identical items by chance. Of all possible items, OSS had 76.4%, WORC 31.2% and SPADI 27.6% identical items.

**Table 1:** Agreement statistics

	Test (SD)	Retest (SD)	Mean difference (95% CI)*	p-value	S_w_	Repeatability coefficient
WORC	44.4 (15.1)	45.0 (16.1)	-0.6 (-3.0 to 1.7)	0.59	6.2	17.2
OSS	43.1 (15.4)	41.3 (12.9)	1.8 (-0.4 to 4.0)	0.11	5.8	16.1
SPADI	51.4 (17.8)	52.6 (18.5)	-1.2 (-3.9 to 1.5)	0.38	7.1	19.7

Agreement estimated by the difference between test and retest, the within-standard deviation (S_w_), and the repeatability coefficient.

* 95% CI For paired t-test under null hypothesis = no difference between test and retest score. 95% CI = 95% confidence interval. SD = standard deviation

**Table 2:** Descriptives and reliability statistics

	Minimum	Maximum	Median	Mean (SD)	IR (%)	ICC (95% CI)	Cronbachs alpha
OSS	15	85	41.7	43.1 (15.4)	7.3	0.83 (0.73 to 0.90)	0.87
SPADI Pain	16	91	58.2	58.9 (17.1)	9.1	0.72 (0.57 to 0.83)	0.74
SPADI Disability	9	85	43.2	44.8 (20.1)	3.6	0.85 (0.76 to 0.91)	0.89
SPADI Total	14	88	49.2	51.4 (17.8)	0.0	0.85 (0.75 to 0.91)	0.91
WORC Physical	21	82	48.9	51.3 (14.9)	3.6	0.78 (0.64 to 0.87)	0.68
WORC Sports	4	86	31.4	34.4 (18.2)	7.2	0.74 (0.59 to 0.84)	0.69
WORC work	3	75	34.0	33.7 (16.7)	5.4	0.75 (0.61 to 0.85)	0.72
WORC lifestyle	10	100 (3.6)	56.1	53.2 (21.5)	7.3	0.82 (0.71 to 0.89)	0.82
WORC emotions	5	98	42.7	47.1 (24.9)	5.5	0.87 (0.81 to 0.93)	0.80
WORC Total	12	77	45.7	44.4 (15.1)	3.6	0.84 (0.75 to 0.91)	0.91

Minimum and maximum scores, median, mean and standard deviations and identical responses on test and retest in percent (IR) of total and domain score for OSS, SPADI and WORC. Reliability statistics estimated by ICC with 95% CI and Cronbachs alpha.

Descriptive data from the first administration of the questionnaires. Retest data is not reported. WORC domain scores presented from 0 (worst possible) to 100 (best possible). The percentage of respondents with maximum score in WORC lifestyle domain is given in parenthesis. 95% CI = 95% confidence interval. SD = standard deviation

The ICC was 0.85 in SPADI, 0.83 in OSS and 0.84 in WORC. The between-scale differences in ICC were small and the confidence intervals relatively wide. The single item ICCs ranged from 0.48 to 0.83 in OSS, 0.52 to 0.90 in WORC and 0.41 to 0.83 in SPADI.

**Figure 1:**
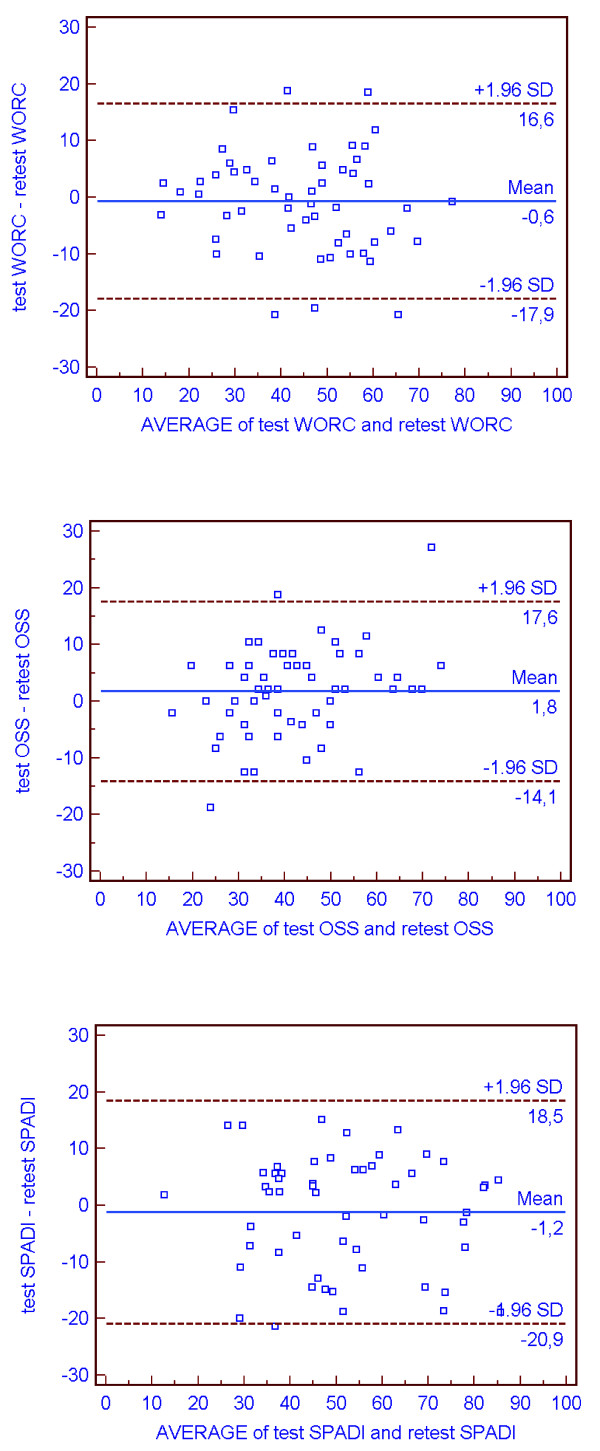
**Limits of agreement plot**. Intraindividual differences (n = 55) between questionnaire responses on test and retest plotted against the mean of the sum scores. On each plot, the central line represents the mean of the intraindividual differences, and the flanking lines represent the 95% limits of agreement.

The within-subject standard deviation, the repeatability coefficients, and the limits of agreement plots are shown in Table 1 and Figure 1, respectively. The internal consistency measured by Cronbach's alpha was high for all total scores and pain and disability domain score in SPADI (Table 2), but WORC physical and sports domain were lower than 0.70. When individual items were deleted values above 0.85 were obtained in all sum scores.

### Validity

Responses from 73 of the 74 patients were used in the validity analysis. One patient left three missing items in the SPADI questionnaire and was therefore excluded from the analysis. The mean age of the 74 patients (26 male, 36%; 47 female, 64%) was 51 years (range 23–80, SD 11). The construct validity was tested by Spearman rank correlations between total and domain sum scores shown in Table 3. The correlations of the total scores were moderate (r = 0.57 to 0.68). As expected, SPADI and OSS sum scores were stronger associated with the pain and physical domain than with the emotions domain of WORC.

**Table 3:** Correlation coefficients

	SPADI pain	SPADI disability	SPADI total	OSS	WORC total
OSS	0.55	0.53	0.57	-	-0.69
WORC Total	-0.60	-0.66	-0.67	-0.69	-
WORC Physical	-0.71	-0.70	-0.75	-0.63	0.82
WORC Sport	-0.43	-0.44	-0.46	-0.49	0.84
WORC Work	-0.50	-0.55	-0.55	-0.64	0.83
WORC lifestyle	-0.57	-0.63	-0.69	-0.61	0.85
WORC emotions	-0.26*	-0,33	-0.31	-0.54	0.76

Spearman rank correlation coefficients showing the strength of the correlations between total and domain scores in OSS, SPADI and WORC.

All correlations were significant on the p < 0.001 level (null hypothesis r = 0.00) except marked *, p < 0.05.

## Discussion

The main findings of the present study were that the agreement, reliability and construct validity of the questionnaires examined are acceptable. Differences in agreement between the questionnaires were small with repeatability coefficients ranging from 16.1% (OSS) to 19.7% (SPADI). A smaller change between two subsequent measurements is indistinguishable from the measurement error and the given limit represents the smallest detectable difference (SDD). The repeatability coefficient for OSS is in agreement with that reported in the original publication. The relatively large variation between two measurements in the same individual should be taken into consideration when assessing follow-up results after treatment and in the planning of prospective studies. Repeated measurements may reduce measurement error and increase the validity of observations.

The estimates of both agreement and reliability parameters reported in this study, supplement current knowledge of clinimetric properties of these shoulder questionnaires. The study was undertaken in a clinical setting including an adequately sized population of patients with rotator cuff disease [25]. We chose to include only patients who evaluated their shoulder condition as unchanged between administrations as measured by the retrospective global change question in the reliability study. The use of retrospective global change questions has been criticized because of the possibility of recall bias [30]. All patients in the present study had experienced shoulder pain for more than 2 months and we did not expect a change in condition within the one-week trial period. A few patients reported relatively large changes in shoulder complaints between administrations. They attributed this to external factors like differences in workload or minor trauma. We believe that excluding these patients reduces the measurement error and is a methodological strength of the study, although the random variation in the clinical setting may be underestimated.

Using the total sum scores of shoulder questionnaires based on ordinal scored items is questionable [31]. As ordinal scales have unknown and unequal intervals, arithmetical or statistical manipulation of the data may be fallacious [32]. These shoulder questionnaires were, however, constructed for using a total sum score and we break the assumptions for statistical operations for pragmatic reasons to get an estimate of the measurement error using the questionnaires as intended. There was a high internal consistency for all scores measured by Cronbach's alpha.

Agreement parameters are expressed on the actual scale of measurement. One unit in OSS may not be directly comparable with one unit on SPADI even when both scores are converted to a score from 0 to 100. Head to head comparisons between agreement parameters of these shoulder scale is therefore difficult. Agreement parameters are however largely independent of the population from which it was determined and therefore comparable between studies [13]. The observed agreement parameter in SPADI was in keeping with the results reported by Schmitt in a mixed population of shoulder and proximal upper arm problems [33], by Angst et al. for patients who had undergone shoulder arthroplasty [34], and somewhat lower than reported by Cloke et al. in a population of patients with impingement syndrome [35]. In concordance with our results, they also reported a lower degree of measurement error in OSS (14.7) than SPADI (20.69). The type of agreement parameter and the sample size may affect the reported values of the measurement error. The value of SEM_agreement _and SEM_consistency _will differ when the systematic differences between test and retest are large [14]. A small sample size may give a biased estimate of the measurement error. The sample size in this study is considered adequate for assessment of the agreement parameter [16].

In the present study, the ICC (2,1) of WORC, OSS and SPADI were 0.84, 0.83 and 0.85 respectively. Arbitrary minimum reliability standards have been recommended by various authors, such as an ICC over 0.70 for questionnaires to be used in group comparison studies [12]. An ICC over 0.90 has been advocated when comparing individuals [12]. According to these standards, the ICCs of the questionnaires are acceptable and better suited for use in group comparisons studies than for evaluation of change in individuals. The lower limit of the relatively wide 95% confidence interval was 0.75 for WORC. A larger sample size would result in narrower confidence intervals that would ease interpretation of the ICCs in relation to the given limits. The ICC of SPADI in the present study was higher than that reported by its developers [20], but in concordance [33] or lower than reported by others [36]. An excellent ICC was reported for WORC (0.96) by the developers of WORC [3]. No reports of ICC of OSS were available in the literature. There is a relatively large difference between ICC of WORC reported in the original study by Kirkley et al. and the results in the present study. It is however important to realize that ICC will be highly dependant on the variation of the study sample and are only generalizable to samples with similar variation. The ICC version used in the study by Kirkley et al. is not described in detail and the choice of ICC could account for some of the differences in results. We must also acknowledge that cultural differences can affect clinimetric properties of questionnaires [12] and that the translation of WORC into Norwegian may not have succeeded in establishing the exact same meaning for each and every item, though the recommendations for cultural adaptations of questionnaires were followed.

Even though SPADI was the most reliable questionnaire according to the point estimate of ICC in this study, the repeatability coefficient of SPADI was highest of the 3 questionnaires, suggesting slightly higher measurement error. This illustrates the need for estimating both reliability and agreement parameters when assessing clinimetric properties in outcome instruments. ICC will increase by extending the variability of the sample, and consequently a heterogeneous population will give a higher ICC than a homogeneous population [12].

Floor and ceiling effects were found for individual items in OSS, WORC and SPADI. Agreement parameters may be overestimated when floor and ceiling effects exists since an extreme value of an item in the test is more likely to be identical on the retest [34]. If floor and ceiling effects are high, extreme items may be missing in the lower or upper end of the scale and patients with the lowest or highest possible score cannot be distinguished from each other, thus reliability is reduced [16]. Responsiveness may also be limited because changes cannot be measured. In the present study, OSS and WORC showed a higher degree of floor and ceiling effects in individual items than SPADI. There were no signs of floor or ceiling effects in total scores in any of the questionnaires, which support the use of these questionnaires to measure change in prospective studies in patients with rotator cuff disease. Further studies should investigate if high floor and ceiling effects in individual items in OSS have consequences for the responsiveness of the questionnaire.

Convergent construct validity was assessed by the Spearman rank correlations coefficient between the total scores of SPADI, OSS and WORC. The strength of the correlations was moderate and lower than expected. If the shoulder instruments were measuring the same constructs, one would expect a strong correlation since the same sample of subjects completed each of the questionnaires. The results of the present study suggest the choice of shoulder questionnaire may have impact on the conclusions made in studies including patients with rotator cuff disease. To assess these implications in longitudinal studies correlation of change in scores should be computed to assess construct validity [18]. Cloke et al. found a higher correlation between OSS and SPADI total scores in patients with impingent syndrome (r = 0.85) than in the present study [35]. Kirkley et al. found a moderate and comparable strength of correlation between the original English WORC and other shoulder and upper extremity questionnaires, ASES (r = 0.68), DASH (r = 0.63), Constant (r = 0.63) and UCLA (r = 0.48) [3].

Future studies should compare the responsiveness of shoulder questionnaires in different clinical settings. Using the minimum detectable difference as an individual threshold and estimate the reliable change proportion would give insightful information when comparing responsiveness between these shoulder questionnaires. Furthermore, an estimation of the minimum clinically important difference would be helpful for proper sample size estimations for future intervention studies.

## Conclusion

We conclude that the agreement and reliability of the three shoulder questionnaires examined, WORC index, SPADI and OSS are acceptable and that differences between scores were small. The Norwegian version of the questionnaires is acceptable for assessing Norwegian-speaking patients with rotator cuff disease. The moderate agreement and construct validity should be taken into consideration when assessing follow-up results after treatment and in the planning of prospective studies.

## Competing interests

The authors declare that they have no competing interests.

## Authors' contributions

JIB, EB–H, OME, EKT and NGJ contributed in the planning process of the present study including the choice of study design, the translational process of the questionnaires and the interpretation of data. OME collected the data. AK and OME performed the statistical analysis. AK contributed to the interpretation of data and helped to draft the manuscript together with JIB, EB–H and OME. All authors read and approved the final manuscript.

## Pre-publication history

The pre-publication history for this paper can be accessed here:


